# Genome-Wide Comparative Analysis of Flowering-Related Genes in Arabidopsis, Wheat, and Barley

**DOI:** 10.1155/2015/874361

**Published:** 2015-09-07

**Authors:** Fred Y. Peng, Zhiqiu Hu, Rong-Cai Yang

**Affiliations:** ^1^Feed Crops Branch, Alberta Agriculture and Forestry, 7000-113 Street, Edmonton, AB, Canada T6H 5T6; ^2^Department of Agricultural, Food and Nutritional Science, University of Alberta, 410 Agriculture/Forestry Centre, Edmonton, AB, Canada T6G 2P5

## Abstract

Early flowering is an important trait influencing grain yield and quality in wheat (*Triticum aestivum* L.) and barley (*Hordeum vulgare* L.) in short-season cropping regions. However, due to large and complex genomes of these species, direct identification of flowering genes and their molecular characterization remain challenging. Here, we used a bioinformatic approach to predict flowering-related genes in wheat and barley from 190 known Arabidopsis (*Arabidopsis thaliana* (L.) Heynh.) flowering genes. We identified 900 and 275 putative orthologs in wheat and barley, respectively. The annotated flowering-related genes were clustered into 144 orthologous groups with one-to-one, one-to-many, many-to-one, and many-to-many orthology relationships. Our approach was further validated by domain and phylogenetic analyses of flowering-related proteins and comparative analysis of publicly available microarray data sets for *in silico* expression profiling of flowering-related genes in 13 different developmental stages of wheat and barley. These further analyses showed that orthologous gene pairs in three critical flowering gene families (PEBP, MADS, and BBX) exhibited similar expression patterns among 13 developmental stages in wheat and barley, suggesting similar functions among the orthologous genes with sequence and expression similarities. The predicted candidate flowering genes can be confirmed and incorporated into molecular breeding for early flowering wheat and barley in short-season cropping regions.

## 1. Introduction

Allohexaploid wheat (*Triticum aestivum* L., 2*n* = 6*x* = 42) and diploid barley (*Hordeum vulgare* L., 2*n* = 2*x* = 14) are two major temperate cereal crop species. The polyploid wheat originated from a two-step natural hybridization of three diploid species, each with seven basic chromosomes (*x* = 7). The first step was the natural hybridization between* Triticum urartu* Tumanian ex Gandilyan (2*n* = 2*x* = 14 AA, the A genome) and* Aegilops speltoides* Tausch (2*n* = 2*x* = 14 BB, the B genome) to form a tetraploid wheat species,* Triticum turgidum* L. [1, 2]. In the second step, the natural hybridization between* Triticum turgidum* L. (2*n* = 4*x* = 28 AABB) and* Aegilops tauschii* Coss. (2*n* = 2*x* = 14 DD, the D genome) occurred to form the hexaploid wheat (AABBDD), which, like many other allopolyploid plant species, has a diploid-like meiotic behavior to prevent the formation of multivalent associations of more than two homologous or homoeologous chromosomes at meiosis [3]. The hexaploid wheat has a very large genome, with an estimated size of about 17 Gb [4] and with more than 80% of the genome consisting of repetitive DNA sequences [5, 6]. Similarly, the diploid barley also has a large genome with an estimated size of about 5.3 Gb and with approximately 84% of the genome being comprised of mobile elements or other repeated structures [7]. Thus, despite recent constructions of physical maps for wheat and barley [6–8], genome-wide characterization of gene functions in these species remains challenging.

Both wheat and barley are widely cultivated mainly for human food, beverages, and animal feed and they are among the top five cereal crops in the world, with a global production of 713 and 145 million tons in 2014 (International Grains Council, http://www.igc.int/en/grainsupdate/sd.aspx). The timing of flowering is one of the most critical agronomic traits influencing grain yield and quality. Early flowering and maturing wheat and barley cultivars are desired in high-latitude regions with short growing seasons and long summer days [9–12]. Additionally, synchronous flowering and maturity can help timely crop harvest to prevent lowered yield and quality due to frost and preharvesting sprouting [13]. Therefore, control of flowering time and the adaptation of flowering to diverse growing environments are vitally important for sustainable production of wheat and barley under changing climate conditions or in different geographical regions.

Most of our understanding of the genetic components and environmental factors triggering floral initiation is gained in the diploid model organism Arabidopsis (*Arabidopsis thaliana* (L.) Heynh., 2*n* = 2*x* = 10), which, like wheat and barley, is a long-day plant, is widely distributed in northern temperate regions, and requires both vernalization (extended exposure to low temperatures) and long photoperiod to stimulate flowering [10, 14–16]. To date, more than 180 genes involved in flowering time control have been identified in Arabidopsis [17–26]. In contrast, only a small number of flowering genes have been studied in temperate cereal crops wheat and barley. These include the pseudoresponse regulator gene* Ppd1* (on 2D) [12, 27–30], TaGI1 (GIGANTEA homolog) [31] and the vernalization genes VERNALIZATION 1 (VRN1) and VRN2 in wheat [15, 32–34], and* Ppd-H1* (on 2H) [35], HvGI [36], HvVRN1 and HvVRN2 [37], HvCO1 (an ortholog of Arabidopsis CONSTANS) [38], EARLY MATURITY 8 (an ortholog of ELF3 in Arabidopsis) [39], and EARLY FLOWERING 3 [40] in barley. Recently, Alqudah et al. [41] compiled a list of 60 genes for their genome-wide association study (GWAS) of photoperiod response in barley. In addition, several reviews about the genetic control of flowering, including those in temperate cereals, have also been published in recent years [10, 16, 42–50], highlighting not only functional conservation but also divergence in molecular mechanisms underlying the floral transition between Arabidopsis and cereal crops. For example, the common ancestor of Arabidopsis and barley is estimated to possess two-thirds of the key circadian clock genes identified in Arabidopsis [51]. The functional orthologs of Arabidopsis CONSTANS (CO) and FLOWERING LOCUS T (FT) have been identified in wheat and barley [21, 48, 52, 53]. However, it is important to note the difference of flowering pathways (most notably the vernalization response) in the dicots and monocots [21]. It should also be recognized that genes with the same name in Arabidopsis and cereals may not be functionally related and vice versa. For example, the* VRN1* gene in wheat and barley is not related to* VRN1* in Arabidopsis but homologous to* AP1/CAL/FUL* [48], and the* VRN3* gene in wheat and barley is an ortholog of* FT* [54].

In addition to experimental identification and characterization of flowering-related genes, computational genomic analysis has become a popular strategy to identify flowering-related genes in economically important crop species, usually using Arabidopsis as the reference. For example, such comparative genomic analyses have been carried out in dicot species including long-day garden pea (*Pisum sativum*) [55], short-day soybean (*Glycine max*) [22, 56], day-neutral mung bean (*Vigna radiata*) [57], and cotton (*Gossypium hirsutum*, cultivated cotton's day-neutral flowering is due to domestication and selective breeding but its wild progenitors require short days) [58], as well as in monocot species including short-day rice [59, 60] and long-day* Brachypodium* (*Brachypodium distachyon*) [61], which is a small temperate grass (purple false brome) with a potential to serve as a new model species for temperate cereal crops [62] and diverged from wheat around 32–39 million years ago (MYA) [63]. These comparative genomic analyses have provided researchers with candidate genes for further molecular characterization to advance our understanding on the genetic control of flowering time in crops. To our knowledge, however, no similar genomic-scale analysis has been reported in wheat; the CCT domain gene family, including CONSTANS-like (COL) and PREUDORESPONSE REGULATOR (PRR) gene families, core circadian clock genes, and a MYB transcription factor (HvLUX1) involved in transcriptional regulation within the circadian clock have been analyzed in barley [51, 64–67].

The genome sequences of bread wheat and barley were released in 2012 [6–8], laying a foundation for identification and comparative analyses of flowering-related genes between Arabidopsis, wheat, and barley on a genome-wide scale. This study has two objectives. The primary objective was to predict putative orthologs of Arabidopsis flowering genes in wheat and barley using a bioinformatic approach that combines reciprocal BLAST searches [68] and OrthoMCL clustering [69, 70]. InterPro domains in all these flowering relevant proteins were compared in Arabidopsis versus wheat or barley [71, 72] and phylogeny analysis was used to validate our approach to ortholog prediction. The secondary objective was to determine whether or not orthologous genes exhibit expression similarities using microarray data analysis. This was achieved by examining gene expression profiles of the flowering genes in different organs and developmental stages using three similar, public transcriptome datasets obtained from the Plant Gene Expression Database PLEXdb [73–76]. Our work was initiated to create a comprehensive collection of flowering-related genes in wheat and barley and their expression profiles in different tissues and developmental stages. This collection will help researchers to select additional genes for further study on genetic control of flowering time in these two important temperate cereal crops.

## 2. Materials and Methods

### 2.1. Identification of Flowering-Related Genes in Wheat and Barley

The 204 flowering genes in Arabidopsis were compiled manually through searches on TAIR [77] and previous studies (Supplemental file 1 in Supplementary Material available online at http://dx.doi.org/10.1155/2015/874361), which include genes with GO (gene ontology) biological process containing one or more terms of circadian rhythm, flowering, flower (floral) development, regulation of flower development, photoperiodism, or vernalization response. The flowering-related genes in wheat and barley were identified using reciprocal BLAST searches followed by OrthoMCL clustering [68–70]. To enable batch BLAST searches, a standalone version of the BLAST tool (version number 2.2.30+) was installed locally and custom search databases were made with its makeblastdb tool. Briefly, a first-round BLAST search (*E*-value < 1*e*-5) was performed using the protein sequences of Arabidopsis flowering genes against the wheat and barley protein databases downloaded from Ensembl (Ensembl Plants release 26, [78]). All the sequences of unique hits in wheat and barley were then used to BLAST against the Arabidopsis proteome (second-round BLAST), and if the original Arabidopsis flowering gene was among the top three hits, the wheat and barley genes were retained as candidate flowering genes. Finally, all proteins of the candidate genes in Arabidopsis, wheat, and barley were subjected to OrthoMCL clustering in OrthoMCL-DB using defaults (*E*-value < 1*e*-5 and match length percentage ≥ 50%) [69, 70]. In the output of OrthoMCL clustering, all flowering proteins were assigned to different orthologous groups (OG), and genes within the same OG as Arabidopsis flowering genes were considered putative orthologs in wheat and barley. OGs with no Arabidopsis flowering genes were excluded.

### 2.2. InterPro Domain Analysis

All protein sequences of the flowering-related genes in Arabidopsis, wheat, and barley were analyzed with a standalone version InterProScan 5 [71, 72]. The default parameters were used, and its InterPro lookup option (iprlookup) was switched on to generate InterPro annotation. For gene encoding multiple proteins (i.e., from alternatively spliced transcripts), its longest sequence was chosen for this analysis.

### 2.3. Multiple Sequence Alignment and Phylogenetic Analysis

Multiple sequence alignment (MSA) was performed using predicted protein sequences with Clustal X (version 2.0) [79] and manually examined with Jalview (version 2.0) [80]. For phylogenetic analysis using a Bayesian approach with BEAST (v1.8.2) [81], input files were first generated using the alignment files from Clustal X (saved as NEXUS format) with BEAUTi (Bayesian Evolutionary Analysis Utility), and the phylogeny was analyzed with BEAST under default settings (1,000,000 generations, four Markov chains, and two runs). The first 25% of the tree from the runs was discarded as burn-in. Then, the tree topology was annotated with TreeAnnotator (both BEAUTi and TreeAnnotator are within the BEAST package). Finally, the phylogenetic tree was viewed with FigTree v1.4.2 (http://tree.bio.ed.ac.uk/software/figtree/). The moss homologs Pp1s34_16V6 (for PEBP) and Phpat.004G002000.1 (for MADS orthogroup OG5_178217) were used as outgroup to root phylogenetic trees.

### 2.4. Expression Analysis of Flowering-Related Genes

The raw data files (.CEL files) for the transcriptome datasets of Arabidopsis, wheat, and barley were retrieved from the plant expression database PLEXdb [76], with experiment AT40 for a gene expression atlas during Arabidopsis development [73], BB3 for transcriptional changes throughout during barley development [74], and TA3 for comparative transcriptomics in the Triticeae [75]. The three raw datasets were analyzed using the same procedure with Bioconductor packages [82] in the open-source statistical R environment [83]. Briefly, the raw data files were imported into Bioconductor using the Simpleaffy package [84] and normalized and transformed to the log_2_ values with the GCRMA package [85]. To get the expression values of flowering genes, each Affymetrix probe set was mapped to an Ensembl gene identifier through BLAST using the flowering gene sequences identified in this study against the target sequences Affymetrix used for the design of these three GeneChips, downloaded from NetAffx Analysis Center (https://www.affymetrix.com/analysis/index.affx). The heatmaps were generated using the heatmap.2 function in the gplots package [83].

## 3. Results

### 3.1. Flowering-Related Genes in Arabidopsis, Wheat, and Barley

Table 1 presents a list of 204 Arabidopsis flowering genes compiled through searches on The Arabidopsis Information Resource (TAIR) and the literature [17, 22, 26, 58, 77]. Of these 204 Arabidopsis flowering genes, 190 genes are known to encode proteins and they were broadly (and somewhat arbitrarily in some cases) classified into seven functional groups as in [22, 56, 58]: autonomous (including ambient temperature pathway), flower development, gibberellin, photoperiod, pathway integration, regulation, and vernalization (see Supplemental file 1 for details). The autonomous pathway consists of genes promoting flowering independently of day-length. The category of flower development includes genes with roles in floral meristem identity and tissue development. The gibberellin (GA) pathway contains genes in GA biosynthesis and metabolism, important for floral transition, and likely inhibits flower formation [86, 87]. The genes in the photoperiod pathway are involved in circadian clock and light signaling. The pathway integration is composed of genes that integrate signals from various flowering pathways. The regulation category contains genes that regulate other flowering genes at transcriptional, posttranscriptional, epigenetic, and posttranslational levels. The vernalization pathway comprises genes for the prolonged exposure of cold temperature required for flowering. The remaining 14 genes are microRNA genes, which are known to regulate the flowering time [88], but these noncoding genes were excluded for subsequent identification of orthologous protein-coding genes in wheat and barley.

A total of 144 distinct ortholog groups (OGs) for all the flowering proteins in these three species were identified (Supplemental file 2). On average, ~1.5 barley and nearly 5.0 wheat copies were identified for each Arabidopsis flowering gene. The identification of the barley gene set may be incomplete [7, 51] and, as a result, the number of orthologous flowering genes we predicted in barley may be underestimated, which is also due to our conservative approach including both reciprocal BLAST searches and OG clustering. In comparison, 491 flowering genes were identified in soybean, a partially diploidized tetraploid, but with a smaller genome size of 1.1–1.15 Gb [22].

As might be expected, complex orthology relationships exist between the flowering genes identified in these three species, including one-to-one, one-to-many, many-to-one, and many-to-many. The vast majority of OGs contain less than 10 genes in Arabidopsis, wheat, and barley, with all OGs containing <10 Arabidopsis flowering genes (Supplemental file 2). A noteworthy exception is OG5_127136 with only one Arabidopsis gene (AT4G39400), but with 75 wheat orthologs and 45 barley orthologs, each of which represents the largest number of flowering genes identified in these two cereal species. The AT4G39400 gene encodes BRASSINOSTEROID INSENSITIVE 1, which is involved in the autonomous pathway that regulates the transition to flowering, mainly through its effects on* FLC* gene expression levels [89]. On the other hand, OG5_139532, an ortholog group (OG) known to contain the soybean FLC [22], includes six Arabidopsis genes: FLOWERING LOCUS C (FLC; AT5G10140), AT1G77080 (MAF1/AGL27), AT5G65050 (MAF2/AGL31), AT5G65060 (MAF3), AT5G65070 (MAF4), and AT5G65080 (MAF5/AGL68). But we only detected one ortholog in barley (MLOC_57890), which matches HvOS1 (ODDSOC1; GenBank accession: HM130526) and HvOS2 (ODDSOC2; HM130525) [90, 91], and wheat (Traes_4AS_E1E60C5E5), which matches TaAGL33 (DQ512366), TaAGL41 (DQ512357), and TaMADS2 (DQ534490) [90]. As in Jung et al. [22], we also tested the whole proteome of wheat and barley (instead of proteins of candidate genes first identified through BLAST analysis, as described in “Section 2” for OrthoMCL clustering, and the results are almost identical, and the total number of OGs containing at least one Arabidopsis flowering gene remains the same (144). This suggests that we have identified the majority of flowering gene orthologs in wheat and barley. This difference between our approach and that of Jung et al. [22] may lead to different false positive and false negative rates in orthology prediction, because using the whole proteome for clustering will likely produce more false orthologs.

Several known flowering genes in wheat and barley have been identified and they offer an opportunity for validation of our approach to ortholog identification. For example, Traes_3B_2A454DB62 and MLOC_68576 represent the FLOWERING LOCUS T (FT) in wheat (TaFT) and barley (HvFT), with the latter already annotated as HvFT in Ensembl. Another example is LFY (AT5G61850), with TaLFY represented by Traes_2AL_83D0D0C3F and Traes_2BL_8DEC0EFBF in wheat and HvLFY represented by MLOC_14305 in barley; all of these three genes have been annotated as LEAFY in the Ensembl database. In addition, Traes_2DS_2A961F39D and MLOC_81154 are putative PPD in wheat (Ppd-D1) and barley (Ppd-H1), respectively. For AtLHY (LATE ELONGATED HYPOCOTYL, AT1G01060), we identified three orthologs in wheat and one in barley (MLOC_14118) (Supplemental file 2). And for* AtCCA1* (CIRCADIAN CLOCK ASSOCIATED 1, AT2G46830), we only predicted one ortholog in barley (MLOC_10707) but not in wheat. Previous studies have shown that one homolog of CCA1/LHY exists in grass species including* Brachypodium*, rice, barley, and wheat [15, 59, 61]. However, discrepancy may exist in our analysis compared with other similar studies, which is generally caused by differences in sequence analysis methods, genomic databases, and parameter settings. For example, Calixto et al. [51] did not find any ortholog of the Arabidopsis* ELF4* gene (*AtELF4*; AT2G40080) in barley and suggested that it might be specific to dicots. However, we identified one putative ortholog each in wheat (Traes_5BL_EC1F3715B1 on chromosome 5B) and barley (MLOC_58590 on 5H; ELF4-like protein annotated by Ensembl), both of which are single-exon genes like* AtELF4*.

During our reciprocal BLAST process (using flowering candidate genes identified in wheat and barley to BLAST the Arabidopsis genome), we identified 101 additional Arabidopsis genes that are related to flowering inferred from sequence similarity (Supplemental file 1). Some of these genes may represent those missed in our manual assembly of Arabidopsis flowering genes based on TAIR and literature searches, while the roles of others in flowering will need to be investigated. Because more than 90% of wheat and barley flowering genes are annotated as “uncharacterized protein” or “predicted protein” in Ensembl (Table 2), we annotated these putative flowering genes identified in wheat and barley (Supplemental file 3), using the annotation of their top BLAST hits in Arabidopsis.

### 3.2. Chromosome Locations of Flowering Genes in Arabidopsis, Wheat, and Barley

The flowering genes do not appear to be randomly distributed on the chromosomes, and flowering gene clusters are noticeable (Supplemental Figure S1). In Arabidopsis, 50 and 58 flowering genes, respectively, are located on the two longest chromosomes (AT1 and AT5). It is known that four MADS Affecting Flowering (MAF) genes (*MAF2*,* MAF3*,* MAF4*, and* MAF5*) are clustered in a ~1.4 Mb (mega base pairs) region on AT5 [92]. In barley, chromosome 2H harbors the most (45) flowering genes, which are mainly located at or near the telomere regions. In wheat, the longest chromosome, 3B, contains the largest number (82) of predicted flowering genes. Nevertheless, since physical positions of all 82 flowering genes on 3B and 293 flowering genes on all other chromosomes were unknown, they were randomly assigned on the respective chromosomes as represented by dashed lines in Figure S1. This lack of information on gene position is caused by the incomplete assembly status of the wheat genome: many assemblies have only been performed to the scaffold level (instead of chromosome level). As a result, only 58% (525/900) of the wheat flowering genes have chromosome positions in the latest GFF3 (General Feature Format for genomic features) file released by Ensembl [78], compared with 97% (265/275) of barley flowering genes and 100% of Arabidopsis flowering genes with chromosome positions. Additionally, the orthologs of flowering genes in wheat are often located on the same group of chromosomes. For instance, the ELF3 (AT2G25930) has three wheat orthologs Traes_1AL_52C5531A4, Traes_1BL_B95F8C666, and Traes_1DL_96D83DE2D, which are located on A1, B1, and D1, respectively. The chromosomal locations of the 101 Arabidopsis genes and their corresponding barley and wheat genes are shown in Supplemental Table S1 and Figure S2.

### 3.3. Exon Intron Organization of Flowering Genes in Arabidopsis, Wheat, and Barley

Motivated by a previous study showing the relationship between gene structure and gene expression in wheat [93], the structural features of the flowering genes in these three species were examined, using the GFF3 files downloaded from Ensembl [78]. As shown in Table 3 (see Supplemental file 4 for details), each Arabidopsis flowering gene has an average of 1.4 transcripts (maximum five transcripts for* LHY* AT1G01060) with a length of 3161 bp. On average, a barley flowering gene has 2.8 transcripts (MLOC_56110 has 27 transcripts, the biggest number of transcripts in barley flowering genes) with an average length of 4328 bp, and a wheat flowering gene only has one transcript (i.e., no alternative splicing) with an average length of 3815 bp. Arabidopsis flowering genes have 6.5 exons on average, with an average length of 466 bp, while barley and wheat have an average number of 4.5 and 5.7 exons with average length of 878 and 565 bp, respectively. The introns are the longest in wheat flowering genes (924 bp), compared with 468 bp in Arabidopsis and 856 bp in barley. On average, the Arabidopsis flowering proteins are the longest (529 amino acids), compared to 444 and 500 in wheat and barley.

Moreover, the intron length variation in VRN-H1 has been shown to affect vernalization sensitivity in barley [94]. We performed a more detailed intron length analysis in the ortholog groups of these flowering genes. Our results show that, overall, genes in wheat and barley have larger intron sizes than their Arabidopsis homologs in the same ortholog group. For example, the OG OG5_170388 includes AP1 (AT1G69120) and CAL/AGL10 (AT1G26310, which is known to be homologous to AP1) with an average of intron length of ~599 bp, nine wheat homologs, with an average intron length of 1761 bp, and one barley gene (MLOC_61901) with average intron length of 2251 bp, which matches VRN-H1 in GenBank (BM5A; AAW82994). However, there are exceptions; in OG5_147177, for example, two Arabidopsis genes (AT1G15550/GA3OX1 and AT1G80340/GA3OX2) have an average intron length of 1598 bp, compared with 486 bp in wheat (six genes: Traes_2AL_B8AB48108, Traes_2BL_9E115B19F, Traes_2BL_FF2BB4801, Traes_2DL_66F9CEA3C, Traes_2DL_F2C4569D7, and Traes_3B_791A6E8DF) and 814 bp in barley (MLOC_12855). Additionally, three OGs (OG5_153242, OG5_160203, and OG5_160330) contain only single-exon genes in all these three species (Supplemental file 4; intron length 0 indicates intronless genes).

### 3.4. Domain Architectures of Flowering Genes in Arabidopsis, Wheat, and Barley

A total of 201 distinct InterPro domains were identified in the flowering proteins of Arabidopsis, wheat, and barley. Among the 144 orthogroups from OrthoMCL clustering, 105 (~91%) OGs (29 OGs with no wheat or barley orthologs excluded) share at least one known InterPro domain (Supplemental file 2). The majority of orthogroups share one or two domains; yet genes in OG5_136555 (an OG known to be involved in light signaling) in these three species share 13 known domains: IPR016132 (Phytochrome chromophore attachment domain), IPR013515 (Phytochrome, central region), IPR003018 (GAF domain), IPR003661 (Signal transduction histidine kinase EnvZ-like, dimerisation/phosphoacceptor domain), IPR001294 Phytochrome, IPR029016 (GAF domain-like), IPR000014 (PAS domain), IPR012129 (Phytochrome A/B/C/D/E), IPR013516 (Phytochrome chromophore binding site), IPR013767 (PAS fold), IPR013654 (PAS fold-2), IPR005467 (Signal transduction histidine kinase, core), and IPR003594 (Histidine kinase-like ATPase, C-terminal domain). This domain analysis further provides confidence in our approach for orthology detection.

**Figure 1:**
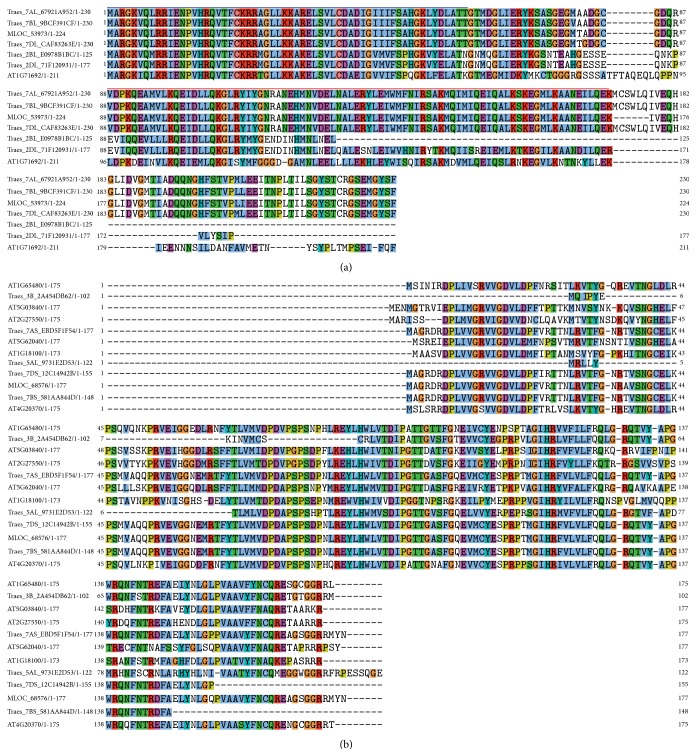
The multiple sequence alignment of OG5_178217 including conserved MADS-box domain and K-box region (a) and PEBP family proteins (b). (a) The MADS-box domain (IPR002100) is marked blue, and the K-box region (IPR002487) marked red. (b) The PEBP domain (IPR008914) in Arabidopsis FT protein encoded by AT1G65480 spans from 27 to 161 amino acids.

The multiple sequence alignments for the MADS-box and PEBP (for phosphatidylethanolamine-binding protein) family proteins show that these domains are more conserved than noncritical regions (Figure 1). The MADS-box near their N-termini is conserved among the genes in the orthogroup OG5_178217, consistent with the fact that the MADS-box is a highly conserved DNA-binding domain; the K-box regions in them are less conserved (Figure 1(a)). In comparison, the PEBP domain is larger (~135 versus <60 aa for MADS) but shows a relatively lower degree of conservation in the proteins of OG5_146543 (Figure 1(b)).

### 3.5. Phylogeny of PEBP and MADS Family Proteins

The plant PEBP gene family shares a PEBP domain (InterPro: IPR00891) and can be classified into three subfamilies: FLOWERING LOCUS T (FT), TFL1 (TERMINAL FLOWER 1), and MFT (MOTHER OF FT). While FT induces flowering, TFL1 suppresses flowering, and MFT mainly regulates seed germination [95, 96]. In Arabidopsis, the PEBP family contains six genes: FLOWERING LOCUS T (FT), TERMINAL FLOWER1 (TFL1), TWIN SISTER OF FT (TSF), BROTHER OF FT AND TFL1 (BFT), CENTRORADIALIS (ATC), and MOTHER OF FT AND TFL1 (MFT). We identified nine PEBP genes in barley and 58 PEBP genes in wheat (Supplemental file 2). Five of the nine barley PEBP genes match those reported in [52, 95]: MLOC_68576 (HvFT) matches HvFT1 (DQ100327), HvFT2 (DQ297407), HvFT3 (DQ411319), and HvFT5 (EF012202); MLOC_13102 and MLOC_71606 are related to HvMFT1 (AB447466); MLOC_74854 is similar to HvFT4 (DQ411320); and MLOC_35818 corresponds to HvTFL1 (AB447465). No HvCEN or HvBFT was reported in [52, 95]. In Arabidopsis, there are 19 flowering genes containing a MADS-box domain, including FLOWERING LOCUS C (*FLC*; AT5G10140) and* MAF2* to* MAF5* (AT5G65050, AT5G65060, AT5G65070, and AT5G65080). We identified eight and 44 MADS proteins in barley and wheat, respectively. Most of these flowering MADS proteins usually also contain a K-box region (IPR002487) near their C-termini (Figure 1(a)).

**Figure 2:**
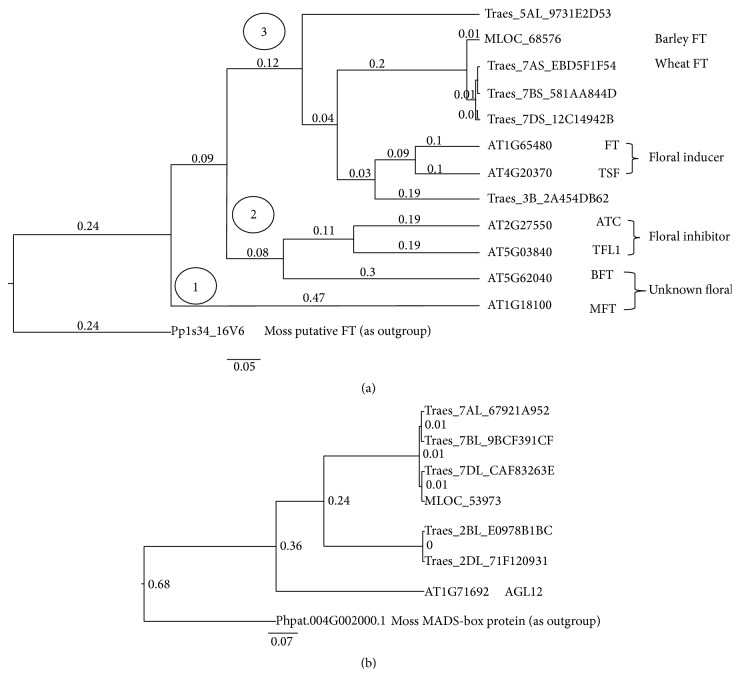
Phylogeny of PEBP (a) and MADS (b) family proteins in Arabidopsis, wheat, and barley. The PEBP proteins include 11 sequences in OG5_146543 (see Supplemental file 2), and a PEBP protein Pp1s34_16V6 in moss was used as an outgroup to root the phylogenetic tree. The MADS AGL12 proteins include six sequences in OG5_178217, and a MADS protein Phpat.004G002000.1 in moss was used as an outgroup to root the phylogenetic tree. The support value on each node is the Bayesian posterior probability. The scale bar denotes the number of nucleotide replacements per site.

In the phylogenetic tree, the three subfamilies are clearly divided into three clades (Figure 2(a)), a topology similar to the phylogenetic relationship of FT proteins in Arabidopsis,* Brachypodium*, rice, and barley previously reported [61]. Interestingly, PEBP genes with known antagonistic roles in flowering responses are in different clades: FT and TSF, two floral inducers, are in one clade, whereas ATC and TFL, two floral inhibitors, are in another. Also, the wheat gene Traes_3B_2A454DB62 is phylogenetically close to AtFT (AT1G65480) and AtTSF (AT4G20370). It is annotated as “uncharacterized protein” in Ensembl, and from our BLAST analysis it is a good hit of Arabidopsis FT (*E*-value = 5.00*e*-48; Supplemental file 3). For the MADS-box proteins, we carried out phylogenetic analysis of OG5_178217, which includes Arabidopsis AGL12 (AT1G71692). As shown in Figure 2(b), two clades were formed, one for AtAGL12 and the other for the five MADS genes in wheat and barley. The latter is further divided into two branches: one for the two wheat genes on the group of 2 chromosomes and the other for four genes on the group of 7 chromosomes (barley MLOC_53973 on 7H).

### 3.6. Expression Profiles of PEBP, MADS, and B-Box Family Genes in Different Organs of Arabidopsis, Wheat, and Barley

**Figure 3:**
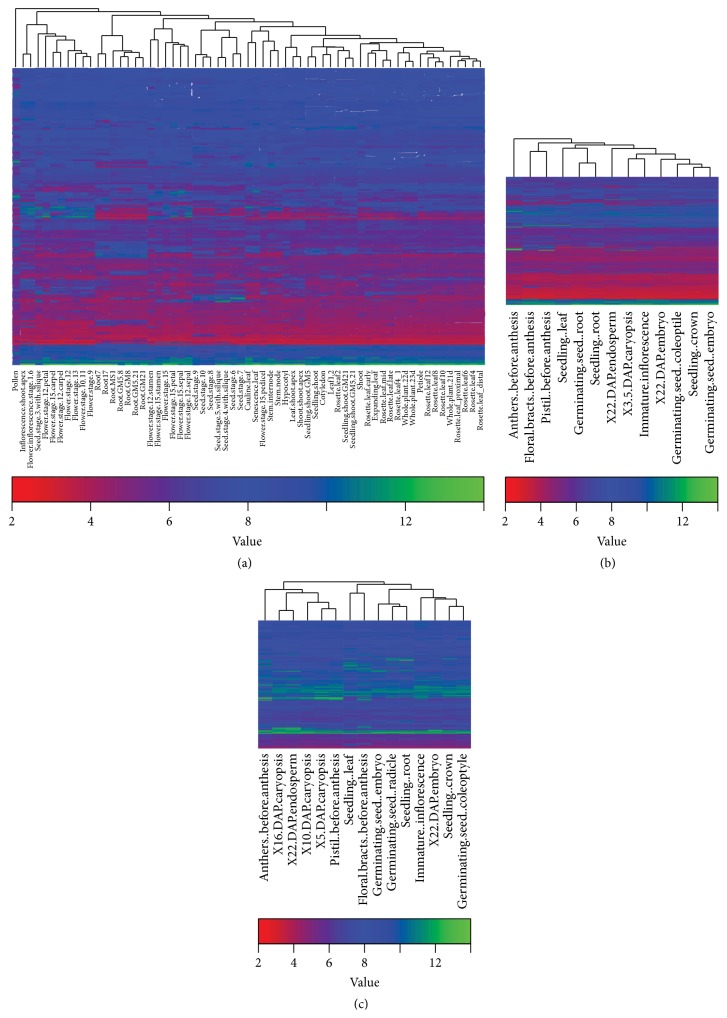
Overview of expression of flowering genes in different tissues and development stages in Arabidopsis (a), wheat (b), and barley (c). The expression data Arabidopsis, wheat, and barley was from 63, 13, and 15 tissue types, respectively (Table 4, Supplemental file 5). The heat maps were created by hierarchical clustering using complete linkage method with the heatmap.2 function in R. The same color key shown is used for all the three heat maps.

Three similar, independent microarray gene expression datasets for Arabidopsis [73], wheat [75], and barley [74] are available in PLEXdb [76], thereby enabling us to analyze the expression profiles of these flowering genes in a wide range of tissues and developmental stages. These three transcriptome datasets were all obtained using the Affymetrix GeneChip platforms and tissues and developmental stages sampled throughout a plant life cycle (Table 4). Additionally, the experimental design of wheat TA3 mirrored that of barley BB3, with 13 of 15 nearly identical tissues [74, 75]. According to our analysis of the 273 raw data files (three replicates for each sample), 189 of 190 Arabidopsis flowering genes were expressed in at least one of the 63 tissues and developmental stages. In barley, 248 (~91%) of the 275 flowering genes are expressed in at least one of the 15 tissue types. Likewise, 676 (~75%) of the 900 putative flowering genes in wheat were expressed in at least one of the 13 tissue types. These percentages for wheat and barley were lower because not all flowering genes we identified were on these two microarrays that were designed using EST (expressed sequence tag) collections (rather than whole genome sequences) in both species (Table 4) [74, 75, 97]. The normalized expression values of flowering genes in Arabidopsis, wheat, and barley are shown in Supplemental file 5. An overview of flowering gene expression in different tissues and development stages of Arabidopsis, wheat, and barley are shown in Figure 3. As evident from the tissue dendrograms, the pollen in Arabidopsis and anthers (before anthesis) in both wheat and barley showed drastically different expression profiles from other tissues. We identified three, 21, and 23 highly expressed flowering genes in Arabidopsis, wheat, and barley, respectively, as represented by the green bands in the heat maps with average log _2_ expression values >10 across all analyzed samples. All three Arabidopsis genes belong to the photoperiod pathway, the green-coded (highly expressed) wheat genes include eight regulatory genes and eight genes in flower development, and the barley green-coded genes include 10 regulatory genes, four photoperiod genes, and five genes related to flower development.

The expression patterns of genes in the different OGs were further compared among the PEBP, MADS-box, and B-box families. These three important gene families contain key genes in the control of flowering time, such as CO (CONSTANS), FLC, FT, FUL (FRUITFULL), and SOC1 (SUPPRESSOR OF OVEREXPRESSION OF CONSTANS1) [23, 95, 96, 98–104]. As no similar Arabidopsis tissues corresponding to those used for expression profiling experiments in wheat and barley were used in the AtGenExpress experiments, we only compared the tissue-specific expression patterns of flowering genes between wheat and barley. Two additional samples (10 DAP caryopsis and 16 DAP caryopsis) exist in barley BB3, which were removed in the barley gene expression dataset in order to compare expression of flowering genes in equivalent tissues of these two cereal species.

**Figure 4:**
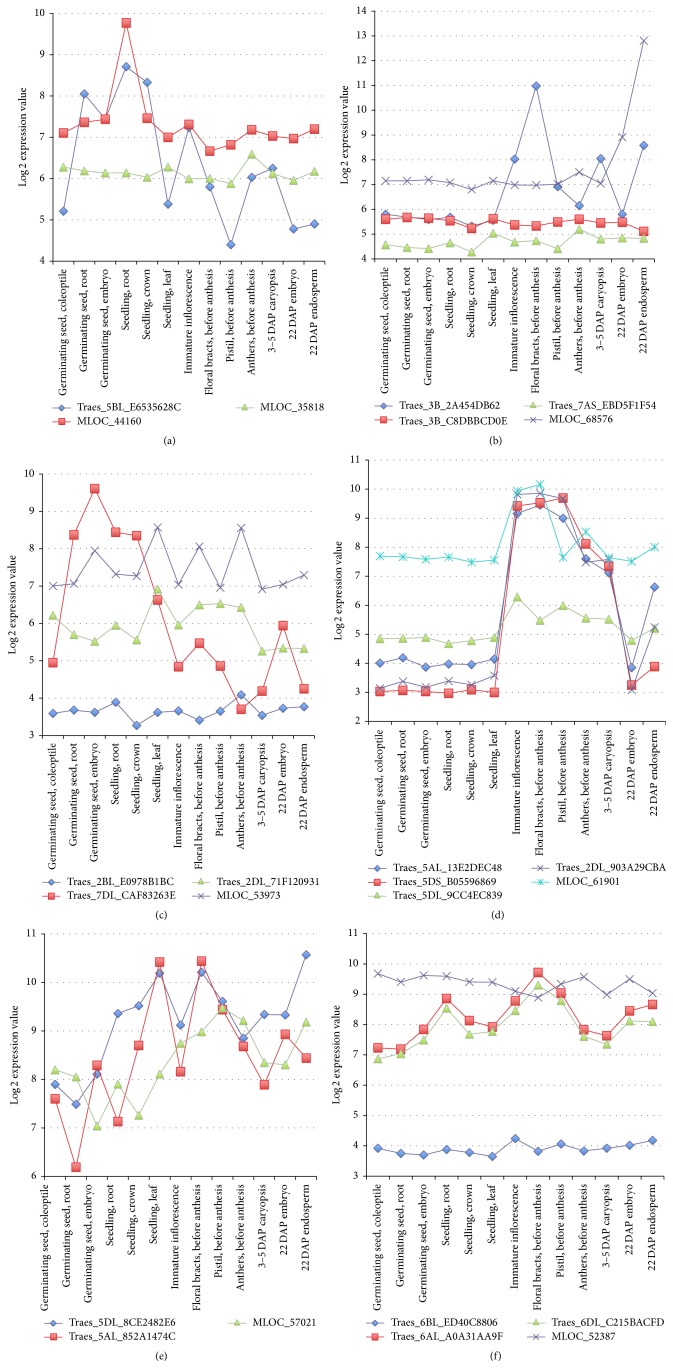
Expression profiles of orthologous genes in the two ortholog groups of the PEBP, MADS-box, and BBX families in wheat and barley. (a): PEBP OG5_158796, (b): PEBP OG5_146543, (c): MADS OG5_178217, (d): MADS OG5_144912, (e): BBX OG5_178368, and (f): OG5_170758.

Figure 4 shows the expression profiles of the major OGs in these three families. The PEBP family proteins were clustered into four OGs: OG5_127642, OG5_146543, OG5_158796, and OG5_163093. Among the three genes in OG5_158796 (Figure 4(a)), Traes_5BL_E6535628C and MLOC_44160 (*HvCEN*) show higher expression in seedling roots, and the other barley gene MLOC_35818 (*HvTFL1*) was relatively weakly and stably expressed in all these tissues. OG5_146543 includes three wheat genes and one barley gene. Traes_3B_2A454DB62 (putative* TaFT*) shows higher expression in immature inflorescence, floral bracts (before anthesis), 3–5 DAP caryopsis, and 22 DAP endosperm, and the barley* FT* gene MLOC_68576 (*HvFT*) has relatively high expression in all the tissues, especially in the 22 DAP endosperm (Figure 4(b)). The comparative expression profiles of additional PEBP genes in ortholog groups OG5_127642 and OG5_163093 are shown in Supplemental Figures S3(A) and S3(B), which also include genes with similar expression patterns, such as Traes_3DS_E0EF3E9AB and MLOC_74854 (*HvFT4*) in OG5_127642.

The MADS-box family of flowering genes was clustered into 11 OGs; yet only six OGs (OG5_135817, OG5_177438, OG5_190130, OG5_144912, OG5_170388, and OG5_178217) have expression data for both wheat and barley flowering genes; OG5_212214, OG5_212591, OG5_139532, OG5_164556, and OG5_211687 have no barley and/or wheat gene expression data for this comparison. Clearly, many MADS flowering genes in each OG show similar expression patterns in the tissues examined in both wheat and barley (Figures 4(c) and 4(d)). For example, the wheat gene Traes_2DL_71F120931 and its barley orthologous gene MLOC_53973 in the ortholog group OG5_178217 exhibit similar expression patterns (Figure 4(c)). Three wheat genes Traes_5AL_13E2DEC48, Traes_5DS_B05596869 (*TaVRN1*), and Traes_2DL_903A29CBA and their barley orthologous gene MLOC_61901 (*VRN-H1*) in OG5_170388 show strikingly similar expression profiles, with elevated expression levels in reproductive tissues including immature inflorescence, floral bracts, pistil, anthers, and 3–5 DAP caryopsis (Figure 4(d)). We also analyzed two microarray datasets after cold and/or light treatments in wheat (NCBI GEO accession: GSE11774) and barley (PLEXdb accession: BB94) and found that both Traes_5DS_B05596869 and MLOC_61901 exhibited an expression profile consistent with that of TaVRN1 and HvVRN1, respectively, as in [86, 105]. Most additional MADS genes in OG5_144912, OG5_177438, OG5_135817, and OG5_190130 also show similar expression profiles (Supplemental Figures S3(C)–S3(F)).

The B-box (BBX) family of transcription factors contains a zinc-finger and B-box domain (IPR000315) with one or two B-box motifs and sometimes also includes a CCT (CONSTANS, CO-like, and TOC1) domain (IPR010402) [104]. The BBX family proteins were clustered into five OGs: OG5_139246, OG5_156319, OG5_178368, OG5_170758, and OG5_170476 (no barley and wheat flowering genes in this OG). The expression profiles of the orthologous BBX genes in OG5_178368 and OG5_170758 are shown in Figures 4(e) and 4(f). Again, similar expression profiles exist in the BBX family genes. The two wheat genes Traes_5DL_8CE2482E6, Traes_5AL_852A1474C and their barley ortholog MLOC_57021 (*HvPRR95*) in OG5_178368 exhibit comparable expression profiles across the 13 tissues (Figure 4(e)). As shown in Figure 4(f), two wheat genes Traes_6AL_A0A31AA9F and Traes_6DL_C215BACFD, as well as their barley orthologous gene MLOC_52387 (*HvTOC1*) in OG5_170758, were all relatively highly expressed in these 13 tissues. Moreover, two wheat genes Traes_2AS_2FCD59730 and Traes_4DL_EE41726EA and the two barley genes MLOC_81154 and MLOC_12732 in OG5_139246, as well as Traes_6DL_036293C55 and MLOC_6921 (putative* HvCO*) in OG5_156319, share similar expression profiles (Supplemental Figures S3(G) and S3(H)). The orthologous genes with similar expression patterns (together with sequence-based homology) in a variety of tissues and development stages are more likely to maintain similar functions related to flowering in wheat and barley.

In addition, when multiple wheat paralogs exist in an OG, some of them are virtually unexpressed (inactive) in the examined tissues. For example, in the PEBP family, both Traes_3B_C8DBBCD0E and Traes_7AS_EBD5F1F54 in OG5_146543 were nearly unexpressed in all these tissues (Figure 4(b)). Both Traes_2BL_E0978B1BC in the MADS family and Traes_6BL_ED40C8806 in BBX family also appear to be unexpressed (Figures 4(c) and 4(f); more examples in Supplemental Figure 3S). Taken together, our expression analysis is consistent with previous studies indicating that homoeologous genes in hexaploid bread wheat can be expressed from one, two, or three homoeoloci [75, 106].

## 4. Discussion

The release of genomic sequences of wheat and barley [6–8] provides a new opportunity for inferring genes and their functions that are agronomically and economically important but yet poorly characterized in these crops through comparative assessment of sequence similarity with the same genes that are well characterized in the model plants. In this study, we used a bioinformatic approach (i.e., reciprocal BLAST searches coupled with OrthoMCL clustering) for identification of putative flowering-related genes in wheat and barley from the known flowering genes in Arabidopsis. Further comparative genomics analyses of these flowering genes in Arabidopsis, wheat, and barley enabled the formation of ortholog groups. Orthologous flowering genes in wheat and barley are often clustered on the same chromosomes, and their exon-intron architectures and key domains are generally conserved.

The intron length of flowering genes in barley and particularly in wheat is generally larger than that of their Arabidopsis homologs (Supplemental file 4), consistent with the comparison of the size of introns in 21 clock genes in Arabidopsis and barley [51]. Szucs et al. [94] showed that the intron length variation in VRN-H1 may account for a continuum of vernalization sensitivity in barley, and thus the consequence of large introns in many cereal genes will need further investigation.

Our domain analysis showed that most of the orthologous flowering proteins share one or more known InterPro domains (Supplemental file 2). As the complete sequence of many cereal genes exhibits low sequence similarity to Arabidopsis genes but shares a higher degree of sequence conservation within protein functional domains [48], domain analysis may play a more important role in prediction of flowering orthologous proteins in monocot crop species.

As our analysis was based on a list of known Arabidopsis flowering genes, we could only find genes with sequence similarity above the threshold in wheat and barley. However, it is known that genes in the vernalization response are not conserved in dicot and monocot between them [48], and our sequence analysis indicates that, compared to those in other pathways, vernalization genes show lowest sequence similarity between Arabidopsis and wheat or barley (Table 5). The parameter setting in reciprocal BLAST [68] and OrthoMCL clustering [69, 70] can also affect the results. In addition, the gene prediction in these two cereal genomes is still incomplete, particularly for barley [7]. As a consequence, our approach shows different performance in different groups of flowering genes. Future studies may focus on a gene family or genes in a flowering pathway, taking into account other sets of genome neighborhood information such as synteny (colinearity), which is particularly important for genes that are less conserved at the sequence level. For example, Ruelens et al. [91] identified two and three FLC-like genes, respectively, in barley and wheat, using an approach that combines phylogenetic reconstruction and genome synteny.

It is evident from our* in silico* expression analyses that many orthologous genes showed similar expression profiles in different tissues of wheat and barley, and sometimes one or more wheat paralogs in an OG were virtually unexpressed in all the thirteen tissues (Figure 4 and Figure S3). These results suggest potential functional conservation and divergence of flowering genes in these two Triticeae species. The unexpressed paralogs in more than 10 developmental stages likely represent pseudogenes.

Several factors affected our* in silico* expression analysis of flowering genes in these species. First, the orthology between barley and wheat genes can be a one-to-many or many-to-many (i.e., not a simple one-to-one) relationship, which can complicate the comparison of their expression profiles. Second, the reliability of gene expression data obtained with the wheat GeneChip can be affected by the fact that wheat is hexaploid with approximately 80% repeats in the genome [6, 75]. Third, as Druka et al. [74] pointed out, the spatial resolution over which they have measured gene expression is low and only a single barley cultivar (Morex) was used. (Similar expression data for the barley cv. Golden Promise can be found but only six tissue types were surveyed.) Lastly and importantly, it would be more interesting to compare the expression profiles of these flowering genes in genotypes with various photoperiod sensitivity and/or vernalization requirements or after different daylength and/or cold temperature treatments, as the expression of many flowering genes is induced by external conditions suitable for flowering [107–109]. For example, two public microarray data sets exist for transcriptomic changes in wheat and barley under the inductive conditions required for flowering [86, 105]; yet the differences in treatments (both cold and light treatments in [86] versus cold treatment in [105]) and tissues sampled (leaf/crown in [86] versus whole plant in [105]) for these two profiling experiments make it difficult to compare the expression patterns of orthologous genes in wheat and barley.

This study has important implication for genetic improvement of early flowering and related traits in wheat, barley, or other cereals. We annotated functions of many flowering-related genes in wheat and barley from known flowering genes identified in Arabidopsis. Of all the annotated genes, those responsible for vernalization and photoperiod are the two most important functional gene groups, accounting for about 70–75% and 20–25% of the genetic variability in the flowering time of wheat, for example, [110, 111]. In western Canada where the growing season for cereal crops is short (95–125 days) with long daylength (>14 h), breeding for early flowering would be most effective with its focus on the use of vernalization genes. For the 20 vernalization genes in Arabidopsis, only eight genes were found in barley and 31 in wheat (cf. Table 1 and Supplemental file 2).

However, while such annotation of vernalization genes is an important first step towards genetic improvement of early flowering in cereal crops, these annotated genes need to be verified before incorporating them into practical breeding programs. Our sequence analysis (Table 5) and several other studies [10, 43, 45, 50] indicated divergence of genes responsible for vernalization response between monocots (e.g., wheat and barley) and dicots (e.g., Arabidopsis). For example, as described earlier, one of the major vernalization genes,* Vrn*2, in wheat and other cereals does not have a clear ortholog in Arabidopsis whereas another main vernalization gene in cereals,* Vrn*1, is homologous to genes that encode proteins APETTALA1 and FRUITFUL with no role in vernalization response in Arabidopsis. For this reason, recent attempts (e.g., [63]) have been made to use phylogenetically more similar cereal species (e.g., rice or* Brachypodium*) as a more immediate model organism for characterization of flowering genes in wheat and barley. However, genetic resources for gene annotation and characterization in rice or* Brachypodium* remain limited in comparison to those in Arabidopsis. Thus, molecular breeding for early flowering and other agronomically important traits in wheat and barley will continue to benefit from comparative genomic analysis with Arabidopsis.

## Supplementary Material

Supplemental file 1: A list of 204 flowering genes and their functional annotations in Arabidopsis compiled through searches in literature and TAIR, and the 101 additional Arabidopsis genes identified in the reciprocal BLAST analysis.
Supplemental file 2: The 144 ortholog groups including genes in Arabidopsis, wheat and barley and their common InterPro domains.
Supplemental file 3: Annotation of the flowering related genes in wheat and barley.
Supplemental file 4: The structural features of the flowering genes in Arabidopsis, wheat and barley.
Supplemental file 5: The expression data of flowering genes in different tissues and developmental stages in Arabidopsis, wheat and barley.
Supplemental Table 1: Distributions of 101 flowering genes over five chromosomes and seven known functional group in Arabidopsis.
Supplemental Figure 1: Distributions of flowering genes in seven functional groups on different chromosomes in Arabidopsis, barley and wheat.
Supplemental Figure 2: Distributions of 101 Arabidopsis flowering genes identified through sequence analysis and their barley and wheat counterparts in different functional groups on different chromosomes.
Supplemental Figure 3: Gene expression profiles in the additional ortholog groups in the PEBP, MADS-box and B-box (BBX) families in different tissues and dvelopment stages in wheat (*Triticum aestivum* cv. Chinese Spring) and barley (*Hordeum vulgare* L. cv. Morex).

## Figures and Tables

**Table 1:** Distributions of 204 flowering genes over five chromosomes and seven known functional groups in Arabidopsis compiled through searches in the literature and TAIR.

Gene type	Functional group	AT1	AT2	AT3	AT4	AT5	Total
Protein coding	Autonomous	0	2	1	3	1	7
	Flower development	19	3	7	3	9	41
	Gibberellin	9	2	3	3	2	19
	Pathway integration	2	1	0	2	2	7
	Photoperiod	9	9	3	1	10	32
	Regulation	8	15	16	10	20	69
	Vernalization	1	1	2	3	8	15
	Subtotal	**48**	**33**	**32**	**25**	**52**	**190**

MicroRNA		2	4	0	2	6	14

	Total	**50**	**37**	**32**	**27**	**58**	**204**

**Table 2:** Flowering-related genes in barley and wheat that are annotated on the basis of top BLAST hits in *Arabidopsis thaliana* expressed as the percentage of characterized and uncharacterized proteins/enzymes in current ENSEMBL annotation.

Ensembl annotation status	New annotation	
	Barley	Wheat
Uncharacterized	93.1% (256)	96.2% (866)
Characterized	6.9% (19)	3.8% (34)

Total	275	900

**Table 3:** Structural characteristics of flowering-related genes in *Arabidopsis thaliana* (AT), *Triticum aestivum* (TA), and *Hordeum vulgare* (HV).

	AT (*n* = 190)		TA (*n* = 525)		HV (*n* = 265)	
	Mean	Range	Mean	Range	Mean	Range
Transcripts per gene	1.4	1–5	1.0	1-1	2.8	1–27
Gene length (bp)	3161	182–16871	3815	240–20952	4328	404–15512
Exons per gene	6.5	1–48	5.7	1–42	4.5	1–20
Exon size (bp)	466	79–4165	565	42–5550	878	87–5211
Intron size (bp)	468	78–2316	924	58–7291	856	44–5912
Protein length (aa)	529	77–3529	444	52–3250	500	50–2056

The numbers of flowering genes used for the summary statistics are shown in parentheses. Single-exon genes (no introns) were excluded for intron size calculation. bp, base pair; aa, amino acid.

**Table 4:** Summary of the three public transcriptome datasets, genome characteristics, and numbers of expressed flowering genes of Arabidopsis, wheat, and barley.

	Arabidopsis	Wheat	Barley
Accession/cultivar	Columbia	Chinese Spring	Morex
Ploidy	Diploid (2*n* = 10)	Hexaploid (2*n* = 6*x* = 42)	Diploid (2*n* = 14)
Genome size	135 Mb	17 Gb	5.3 Gb
Number of total predicted genes	27,416	9,8897	24,287
Number of genes on GeneChip	22,814	61,290	22,840
PLEXdb experiment ID	AT40	TA3	BB3
Number of tissues sampled^a^	63	13	15
Number of predicted flowering genes	190	900	273
Number of flowering genes expressed (% of predicted flowering genes)	189 (99%)	676 (75%)	248 (91%)

^a^Tissues of wheat include germinating seed (coleoptile, root, and embryo), seedling (root, crown, and leaf), immature inflorescence, floral organs before anthesis (bracts, pistils, and anthers), 3–5 DAP caryopsis, 22 DAP embryo, and 22 DAP endosperm; tissues of barley include germinating seed (coleoptile, root, and embryo), seedling (root, crown, and leaf), immature inflorescence, floral organs before anthesis (bracts, pistils, and anthers), 3–5 DAP caryopsis, 10 DAP caryopsis, 16 DAP caryopsis, 22 DAP embryo, and 22 DAP endosperm. See Supplemental file 5 for the normalized, log2-transformed expression values of the flowering genes in different organs or developmental stages.

**Table 5:** The average percentage of protein sequence similarity of flowering genes in the seven functional groups in Arabidopsis, wheat, and barley.

Functional group	AT versus TA	AT versus HV	HV versus TA
Autonomous	62.72 (16.91)	53.36 (14.48)	97.32 (2.28)
Flower development	68.77 (11.83)	63.23 (12.39)	95.19 (6.21)
Gibberellin	61.27 (15.07)	54.71 (10.95)	96.85 (2.01)
Pathway integration	74.75 (13.39)	58.95 (16.97)	95.44 (3.81)
Photoperiod	61.44 (17.99)	57.37 (16.25)	94.48 (5.99)
Regulation	69.78 (13.04)	61.97 (13.49)	96.49 (3.75)
Vernalization	58.88 (8.31)	44.63 (13.04)	81.64 (15.04)

AT versus TA, comparison of flowering protein sequences between Arabidopsis and wheat; AT versus HV, comparison of flowering protein sequences between Arabidopsis and barley; HV versus TA, comparison of flowering protein sequences between barley and wheat. The values in the parentheses are standard deviations.
