# Supplementary material for: Genome-Wide Comparative Analysis of Flowering-Related Genes in Arabidopsis, Wheat, and Barley
Source: Int J Plant Genomics. 2015 Sep 7;2015:874361. doi: 10.1155/2015/874361 (PMC4576011; doi:10.1155/2015/874361)
Supplement: Supplementary file 1 — Supplemental file 1: A list of 204 flowering genes and their functional annotations in Arabidopsis compiled through searches in literature and TAIR, and the 101 additional Arabidopsis genes identified in the reciprocal BLAST analysis. Supplemental file 2: The 144 ortholog groups including genes in Arabidopsis, wheat and barley and their common InterPro domains. Supplemental file 3: Annotation of the flowering related genes in wheat and barley. Supplemental file 4: The structural features of the flowering genes in Arabidopsis, wheat and barley. Supplemental file 5: The expression data of flowering genes in different tissues and developmental stages in Arabidopsis, wheat and barley. Supplemental Table 1: Distributions of 101 flowering genes over five chromosomes and seven known functional group in Arabidopsis. Supplemental Figure 1: Distributions of flowering genes in seven functional groups on different chromosomes in Arabidopsis, barley and wheat. Supplemental Figure 2: Distributions of 101 Arabidopsis flowering genes identified through sequence analysis and their barley and wheat counterparts in different functional groups on different chromosomes. Supplemental Figure 3: Gene expression profiles in the additional ortholog groups in the PEBP, MADS-box and B-box (BBX) families in different tissues and dvelopment stages in wheat (Triticum aestivum cv. Chinese Spring) and barley (Hordeum vulgare L. cv. Morex). [file 874361.f1.zip › Supplemental file 6.pdf]

Supplemental Table S1 - Distributions of 101 flowering genes over five chromosomes and seven known functional group in Arabidopsis.

Functional group	AT1	AT2	AT3	AT4	AT5	Total
Autonomous	10	1	4	4	8	27
Flower development	7	1	6	2	4	20
Gibberellin	3	1	2	2	3	11
Pathway integration	1	0	0	0	0	1
Photoperiod	3	1	3	4	4	15
Regulation	1	8	4	5	3	21
Vernalization	0	1	1	2	2	6
Total	25	13	20	19	24	101

Supplemental Figure S1: Distributions of flowering genes in seven functional groups on different chromosomes in Arabidopsis, barley and wheat.

AT1 – AT5 for the five chromosomes in Arabidopsis; HV1 – HV7 for the seven chromosomes in barley; TA1 – TA7 for the seven chromosomes in the A genome of wheat; TB1 – TB7 for the seven chromosomes in the B genome of wheat; TD1 – TD7 for the seven chromosomes in the D genome of wheat. Flowering genes without physical locations on wheat chromosomes are randomly assigned and represented by dashed lines.

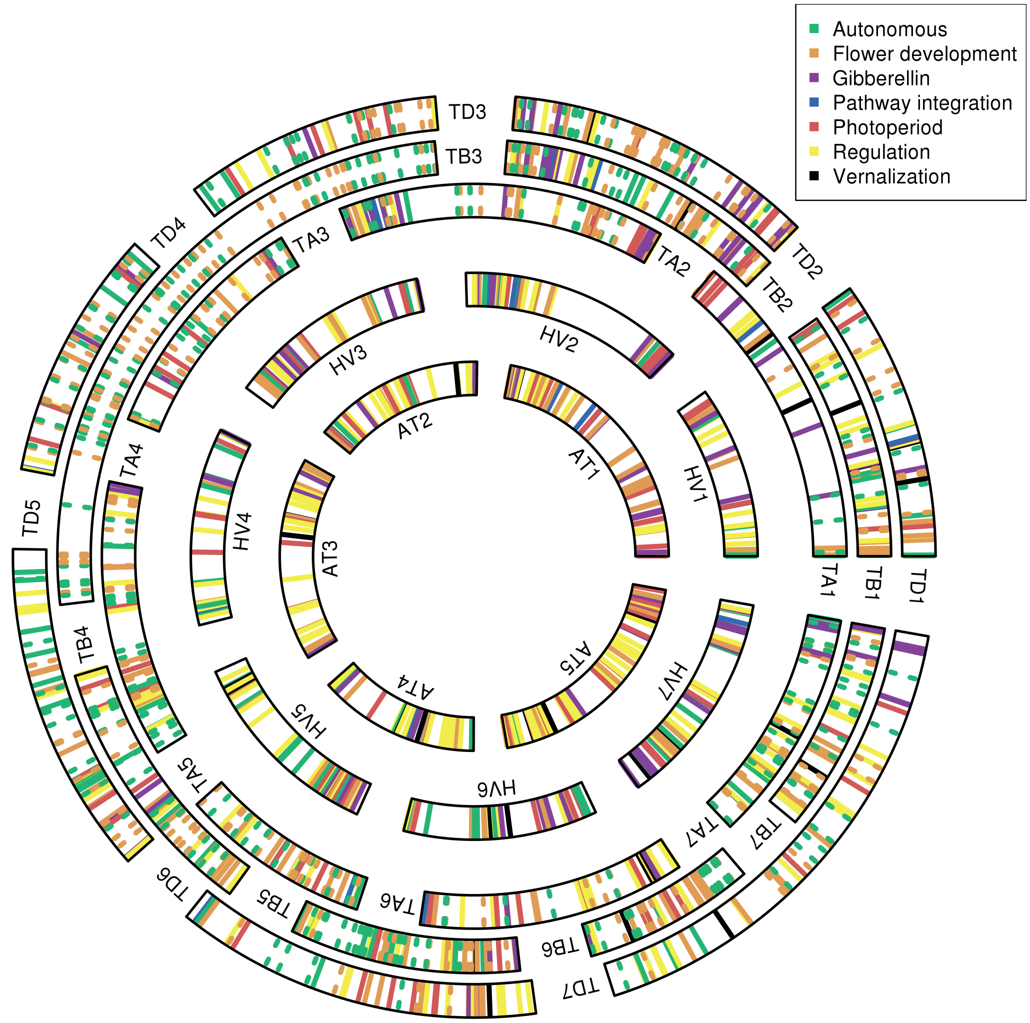

Supplemental Figure S2: Distributions of 101 Arabidopsis flowering genes identified through sequence analysis and their barley and wheat counterparts in different functional groups on different chromosomes.

AT1 – AT5 for the five chromosomes in Arabidopsis; HV1 – HV7 for the seven chromosomes in barley; T1A – T7A for the seven chromosomes in the A genome of wheat; T1B – T7B for the seven chromosomes in the B genome of wheat; T1D – T7D for the seven chromosomes in the D genome of wheat. Flowering genes without physical locations on wheat chromosomes are represented by dashed lines.

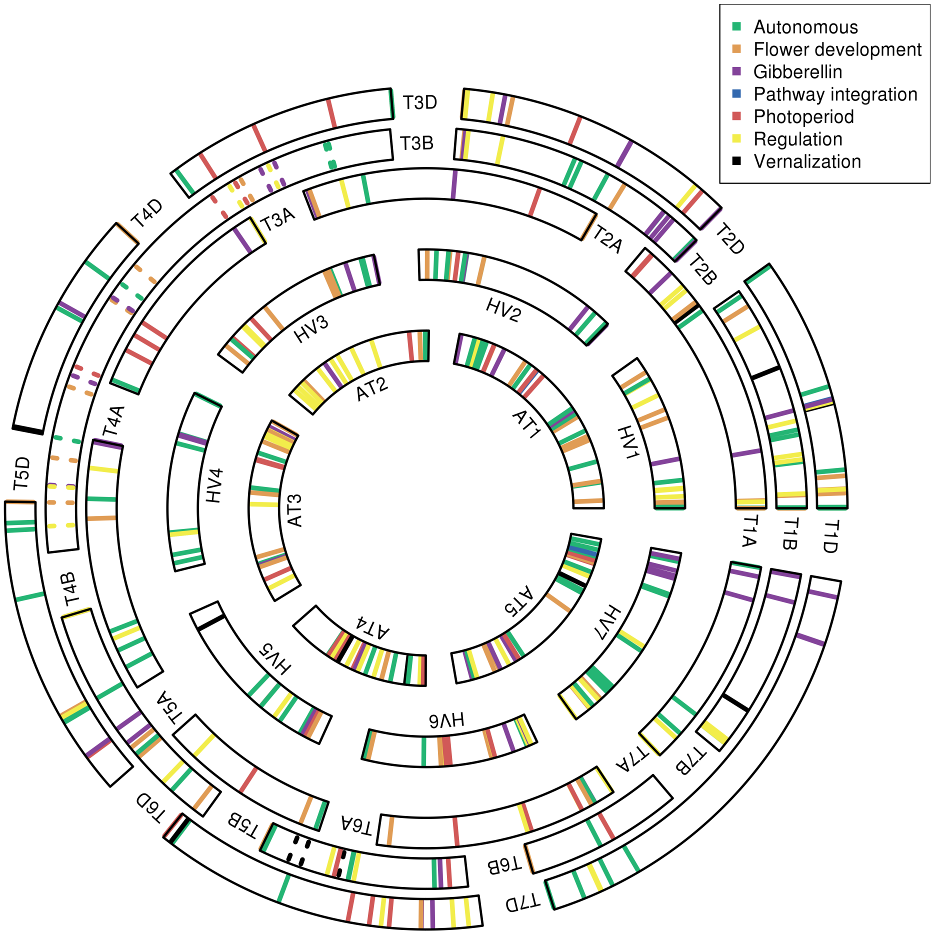

Supplemental Figure S3: Gene expression profiles in the additional ortholog groups in the PEBP, MADS-box and B-box (BBX) families in different tissues and dvelopment stages in wheat (*Triticum aestivum* cv. Chinese Spring) and barley (*Hordeum vulgare* L. cv. Morex).

A) OG5_127642, and B) OG5_163093 in the PEBP family; C) OG5_144912, D) OG5_177438, E) OG5_135817, and F) OG5_190130 in the MADS-box family; G) OG5_139246 and H) OG5_156319 in the BBX family.

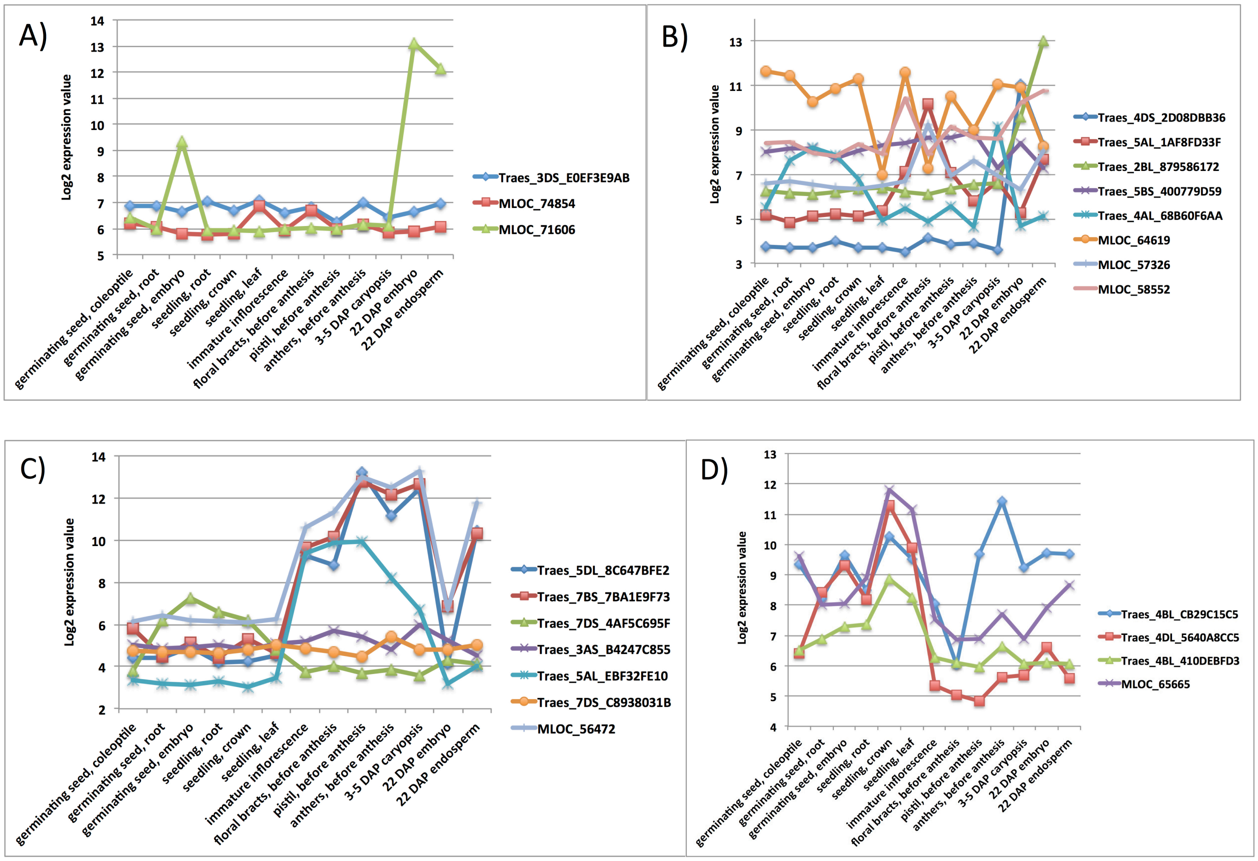

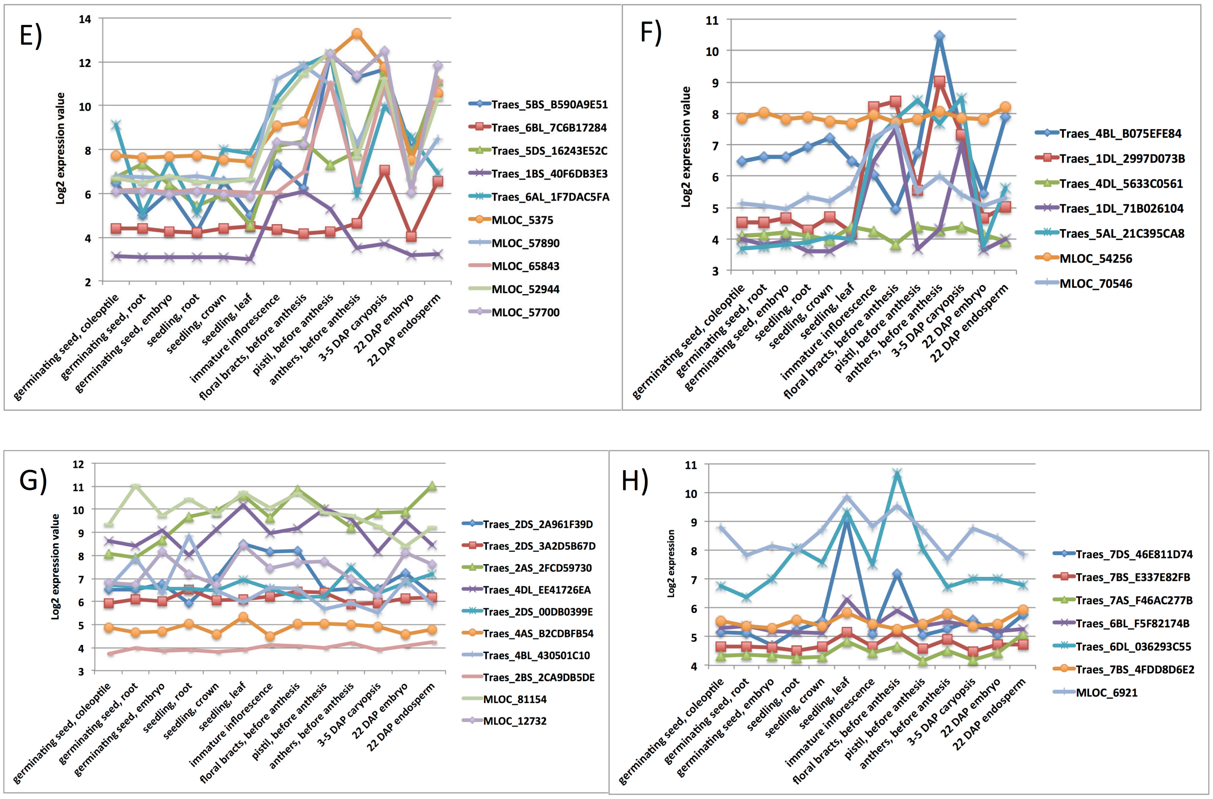
E)
Log2 expression value
Traes_5BS_B590A9E51
Traes_6BL_7C6B17284
Traes_5DS_16243E52C
Traes_1BS_40F6DB3E3
Traes_6AL_1F7DAC5FA
MLOC_5375
MLOC_57890
MLOC_65843
MLOC_52944
MLOC_57700
F)
Log2 expression value
Traes_4BL_B075EFE84
Traes_1DL_2997D073B
Traes_4DL_5633C0561
Traes_1DL_71B026104
Traes_5AL_21C395CA8
MLOC_54256
MLOC_70546
G)
Log2 expression value
Traes_2DS_2A961F39D
Traes_2DS_3A2D5B67D
Traes_2AS_2FCD59730
Traes_4DL_EE41726EA
Traes_2DS_00DB0399E
Traes_4AS_B2CDBFB54
Traes_4BL_430501C10
Traes_2BS_2CA9DB5DE
MLOC_81154
MLOC_12732
H)
Log2 expression
Traes_7DS_46E811D74
Traes_7BS_E337E82FB
Traes_7AS_F46AC277B
Traes_6BL_F5F82174B
Traes_6DL_036293C55
Traes_7BS_4FDD8D6E2
MLOC_6921
germinating seed, coleoptile
germinating seed, root
germinating seed, embryo
seedling, root
seedling, crown
seedling, leaf
immature inflorescence
floral bracts, before anthesis
pistil, before anthesis
anthers, before anthesis
3-5 DAP caryopsis
22 DAP embryo
22 DAP endosperm
